# *Ribes fasciculatum* Ameliorates High-Fat-Diet-Induced Obesity by Elevating Peripheral Thermogenic Signaling

**DOI:** 10.3390/molecules27051649

**Published:** 2022-03-02

**Authors:** Yuna Lee, Yeo-Jin Park, Bonggi Lee, Eunkuk Park, Hail Kim, Chun-Whan Choi, Min-Soo Kim

**Affiliations:** 1 Brain Science Institute, Korea Institute of Science and Technology (KIST), Seoul 02792, Korea; yuna.lee@kist.re.kr; 2 Division of Bio-Medical Science & Technology, KIST School, University of Science and Technology, Seoul 02792, Korea; 3 Korean Medicine (KM) Application Center, Korea Institute of Oriental Medicine, Daegu 41062, Korea; pyjin5526@kiom.re.kr; 4 Korean Convergence Medicine, University of Science and Technology, Daejeon 34054, Korea; 5 Department of Food Science and Nutrition, Pukyong National University, Busan 48513, Korea; bong3257@pknu.ac.kr; 6 Department of Medical Genetics, Ajou University School of Medicine, Suwon 16499, Korea; jude0815@hotmail.com; 7 Graduate School of Medical Science and Engineering, Korea Advanced Institute of Science and Technology, Daejeon 34504, Korea; hailkim@kaist.edu; 8 Natural Product Research Team, Gyeonggi Biocenter, Gyeonggido Business and Science Accelerator, Suwon 16229, Korea

**Keywords:** *Ribes fasciculatum*, obesity, thermogenesis, afzelin

## Abstract

*Ribes fasciculatum* has been consumed as a food and as a traditional medicine for treating autoimmune diseases and aging in diverse countries. A previous study showed that a mixture of *Ribes fasciculatum* and *Cornus officinalis* prohibited adipocyte differentiation and lipid accumulation in preadipocytes and suppressed diet-induced obesity. Nevertheless, the mechanism of *R. fasciculatum* to regulate energy homeostasis solely through thermogenic signaling remains unclear. Thus, we investigated its effects on energy homeostasis using *R. fasciculatum* fed to C57BL/6 mice with a 45% high-fat diet. Chronic consumption of *R. fasciculatum* decreased the body weight of obese mice with increasing food intakes and improved metabolic-syndrome-related phenotypes. Therefore, we further tested its thermogenic effects. Cold chamber experiments and qPCR studies indicated that *R. fasciculatum* elevated thermogenic signaling pathways, demonstrated by increased body temperature and uncoupling protein 1 (UCP1) signaling in the white and brown adipose tissues. Afzelin is one major known compound derived from *R. fasciculatum*. Hence, the isolated compound afzelin was treated with preadipocytes and brown adipocytes for cell viability and luciferase assay, respectively, to further examine its thermogenic effect. The studies showed that the response of afzelin was responsible for cell viability and the increased UCP1. In conclusion, our data indicated that *R. fasciculatum* elevated peripheral thermogenic signaling through increased UCP1 via afzelin activation and ameliorated diet-induced obesity.

## 1. Introduction

Obesity has been a significant global public health concern due to excessive energy storage. Thus, its association with various metabolic disorders, such as cardiovascular diseases, hypertension, diabetes, and fatty liver, has led to the development of numerous anti-obesity therapies [1].

Among the various anti-obesity approaches, drug therapy is a widely used strategy for severe obesity patients [2]. Nevertheless, despite the efficacy of drugs, many anti-obesity treatments have not been successful because of adverse side effects [2]. For example, the negative effects of orlistat, which is the only long-term obesity treatment being applied, were shown to be moderately unfavorable [3]. In this situation, natural products and compounds isolated from them have been attractive candidates for a safer therapeutic strategy for obese individuals.

Additionally, brown adipose tissue (BAT) plays a role in heat generation and body temperature maintenance through burning energy. Moreover, the anti-obese effect of thermogenesis stimulation, in which white adipocytes are converted to beige adipocytes, also known as browning white adipose tissue (WAT), is a concern of interest. Peripheral factors, including retinoblastoma interaction with the zinc finger protein homology domain, which contains 16, exercise, irisin, and norepinephrine; β3-adrenergic receptors; thyroid prohormone thyroxine (T4); chronic cold exposure; norepinephrine; and peroxisome proliferator-activated receptor γ (PPARγ) agonists, activate BAT and browning of WAT [4,5]. In addition, the hypothalamus, the central nervous system that regulates thermogenesis and energy homeostasis, can control WAT browning and BAT activation by acquiring a signal of sympathetic nerve activity [6]. Evidence shows that increasing energy expenditure with WAT browning and BAT activation is an effective pharmaceutical mechanism for anti-obesity and anti-metabolic-syndrome effects [5,7,8].

*Ribes fasciculatum* var. chinense MAX. (Saxifragaceae) is widely cultivated in diverse countries, including Korea, Japan, and China [9,10,11]. Although only a few biological activities are known, *R. fasciculatum* (RF) has been applied for individuals with autoimmune diseases and aging-related disorders [11,12]. RF extract was reported to possess neuroprotective effects in hydrogen-peroxide-induced SH-SY5Y cells and memory-impaired SD rats with scopolamine [13,14]. In addition, mixed extracts of RF and *Cornus officinalis* have shown anti-adipogenesis effects in 3T3-L1 cells, preadipocytes, high-fat diet (HFD)-fed mice, and overweight women [15,16]. Thus, RF is considered a potential natural source of anti-obesity products.

Recently, our groups identified afzelin, a major compound of RF. We identified 11 substances isolated from RF, with being afzelin one of them. Moreover, we reported that afzelin is a significant compound with neuroprotective and cognition-improving effects in a dementia mouse model [17]. Hence, this study will examine the anti-obesity effects of RF and its mechanisms.

## 2. Results

### 2.1. RF Decreased HFD-Induced Obesity

**Figure 1:**
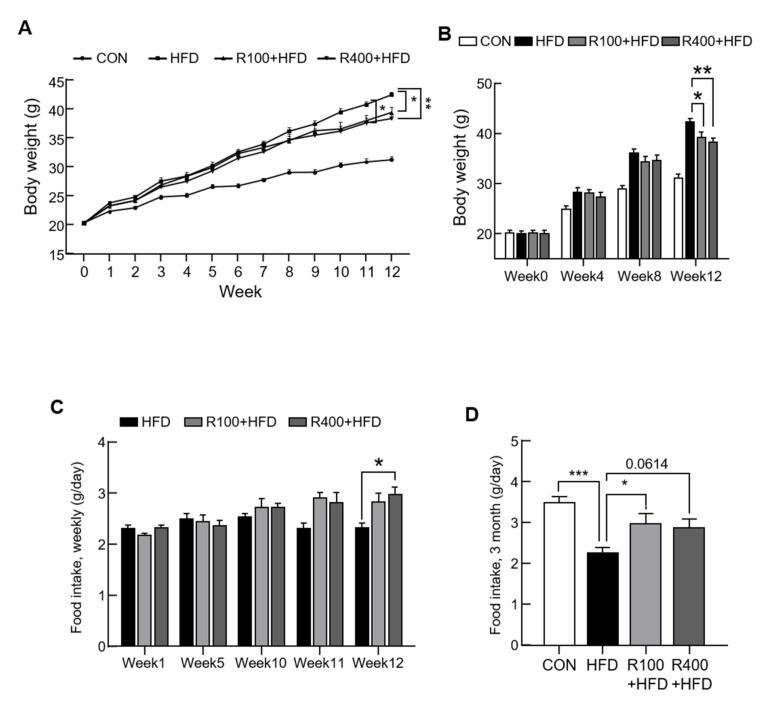
Anti-obesity effect of *R. fasciculatum* extracts. Standard C57BL/6 mice (5 weeks old) were fed with chow diet (CON), high-fat diet (HFD, 45 kcal% fat diets), 100 mg *R. fasciculatum* (RF) extract/HFD kg (R100 + HFD), or 400 mg RF/HFD kg (R400 + HFD) for 12 weeks. (**A**,**B**) Body weight was measured every week for 12 weeks (*n* = 7–8). (**C**) Diet was provided and weighed every week. The amount of weekly food intake was divided by 7 (*n* = 4–6). (**D**) Mice fasted for 8 h and were placed in individual cages for the experiment. Daily food intake was measured at 3 months (*n* = 6–7). Data expressed as mean ± standard error of the mean (S.E.M.). Statistical differences were determined using one-way ANOVA, followed by Tukey’s post-hoc test: * *p* < 0.05, ** *p* < 0.01, *** *p* < 0.001.

Diets mixed with RF extracts (100 or 400 mg/kg) and HFDs (45% of calories as fat, HFD) were produced to examine the energy-balance-regulating effect of the RF extracts. Mixed diets, low-dose RF (100 mg/kg, R100 + HFD), or high-dose RF (400 mg/kg, R400 + HFD) were also administered to normal C57BL/6 mice, and their body weight and food intake were monitored for 3 months. Expectedly, the group fed with RF extracts at both a low and a high dose (100 and 400 mg/kg, respectively) showed significantly reduced body weight gain compared with the HFD-fed group (Figure 1A,B). We measured all food intakes for 3 months to evaluate if the RF-mediated body weight gain alteration was due to the change in feeding behavioral change. Interestingly, the food intake of the R400 + HFD group gradually increased, with a significant difference from the HFD group at 12 weeks (Figure 1C). When we measured the control group’s food intake at 3 months, the RF groups showed an increased food intake level (Figure 1D), similar to the weekly food intake. This observation suggested that RF extracts controlled the food intake signaling, consequently decreasing body weight gain.

### 2.2. Ribes fasciculatum Ameliorates Blood Metabolic Parameters

**Figure 2:**
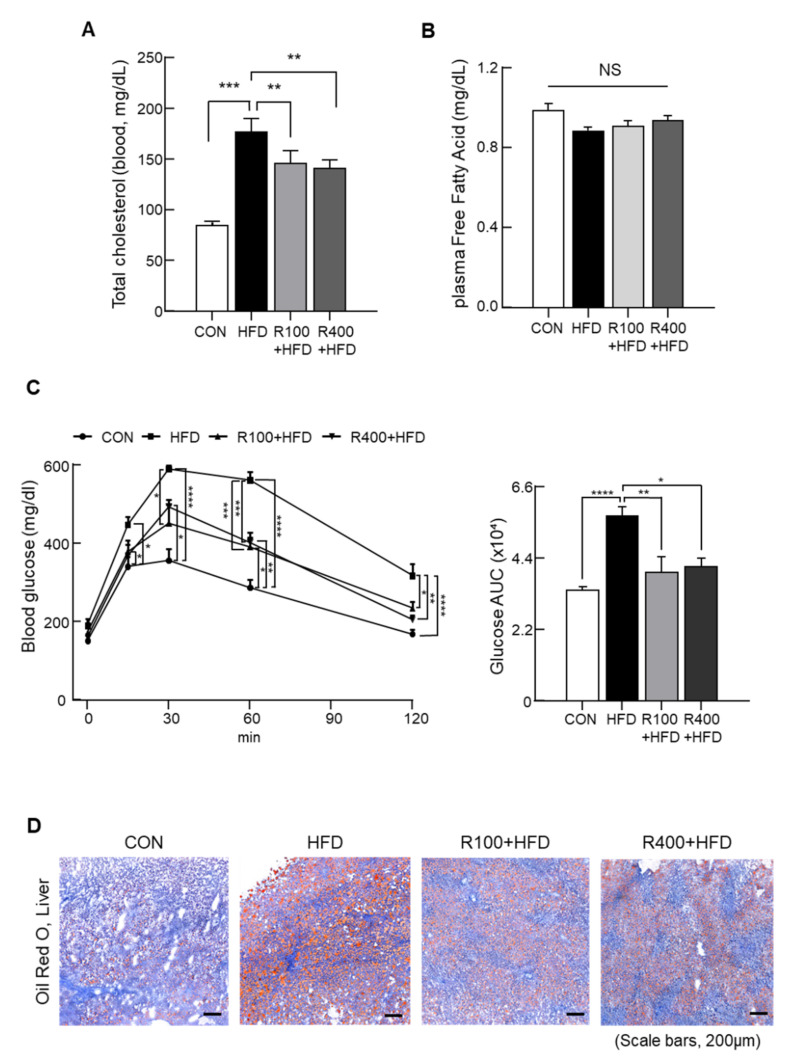
Improvement of blood metabolic parameters by *R. fasciculatum.* C57BL/6 mice (5 weeks old) were fed with chow diet (CON), high-fat diet (HFD, 45 kcal% fat diets), 100 mg *R. fasciculatum* (RF)/HFD kg (R100 + HFD), or 400 mg RF/HFD kg (R400 + HFD). The blood of these mice was collected at least 8 h after fasting to evaluate blood metabolic parameters. (**A**) Total cholesterol (*n* = 7–8) in the plasma was measured using pharmaceutical enzymatic kits. (**B**) Free fatty acid (*n* = 5–6) in plasma was analyzed with acyl-CoA synthetase–acyl-CoA oxidase (ACS-ACOD) with the NEFA-HR (non-esterified fatty acids) reagent. (**C**) Glucose tolerant test was performed at 3 months after 8 h fasting (*n* = 3–7). The area under the curve was calculated from these data. (**D**) Oil red O staining showed fat deposition in the liver (*n* = 4). Data expressed as mean ± standard error of the mean (S.E.M.). Statistical differences were determined using one-way ANOVA, followed by Tukey’s post-hoc test: * *p* < 0.05, ** *p* < 0.01, *** *p* < 0.001, **** *p* < 0.0001.

Blood metabolic parameters, blood glucose, and lipid profile were measured to determine the association with RF-induced body weight reduction. The total cholesterol (TC) increase in HFD was suppressed significantly by both doses of the RF mixed diet (Figure 2A). Additionally, there was no significant alteration in the blood free fatty acid (FFA) level at that intake of RF (Figure 2B). The glucose tolerance test revealed improved glucose tolerance in the R100 + HFD and R400 + HFD group from 30 to 120 min compared with the HFD group (Figure 2C). In the liver, oil red O staining showed a reduction of hepatic lipid droplets at both doses of RF uptake, demonstrating the improvement of fatty acids metabolism by RF feed (Figure 2D). These data indicate that the body-weight-lowering effect of RF is related to the enhancement of metabolic parameters.

### 2.3. RF Control Energy Expenditure by Elevating Body Temperature

**Figure 3:**
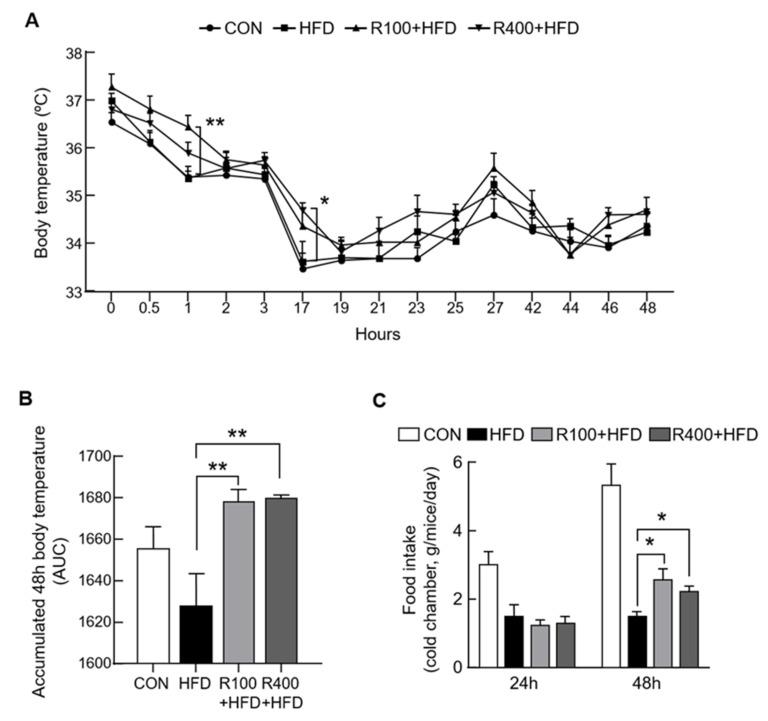
Feeding the *R. fasciculatum* mixed diet improved cold resistance. Standard C57BL/6 mice (5 weeks old) were fed with chow diet (CON), high-fat diet (HFD, 45 kcal% fat diets), 100 mg/kg of *R. fasciculatum* (RF) extract mixed with HFD (R100 + HFD), or 400 mg/kg of *R. fasciculatum* (RF) extract mixed with HFD (R400 + HFD) for 12 weeks. (**A**) Body temperatures were measured using a rectal thermometer (*n* = 5–7). (**B**) Body temperature at 48 h. The area under the curve was calculated using values from (**A**) (*n* = 5). (**C**) Food intake was weighed at 24 and 48 h during the cold resistance test (*n* = 5–8). Data are expressed as mean ± standard error of the mean (S.E.M.). Statistical differences were determined using one-way ANOVA, followed by Tukey’s post-hoc test: * *p* < 0.05, ** *p* < 0.01.

It is hypothesized that RF may alter energy expenditure, since RF reduces weight gain despite increased food intake. Thus, a cold challenge test was performed to determine this effect. First, the experimental mice were placed in a cold chamber (4 °C); then, the rectal temperature as well as 24 h and 48 h food intake were measured. The data showed that the rectal body temperature was higher in both mice groups fed with the RF mixed diet than the HFD group (Figure 3A,B). Furthermore, the dietary intake of R100 + HFD and R400 + HFD groups in the cold chamber significantly increased compared with HFD groups at 48 h (Figure 3C). From these results, it may be suggested that the increased energy consumption due to thermogenesis contributes to the reduction in weight gain in the RF groups.

### 2.4. Oral RF Feeding Induces Thermogenesis Signals

**Figure 4:**
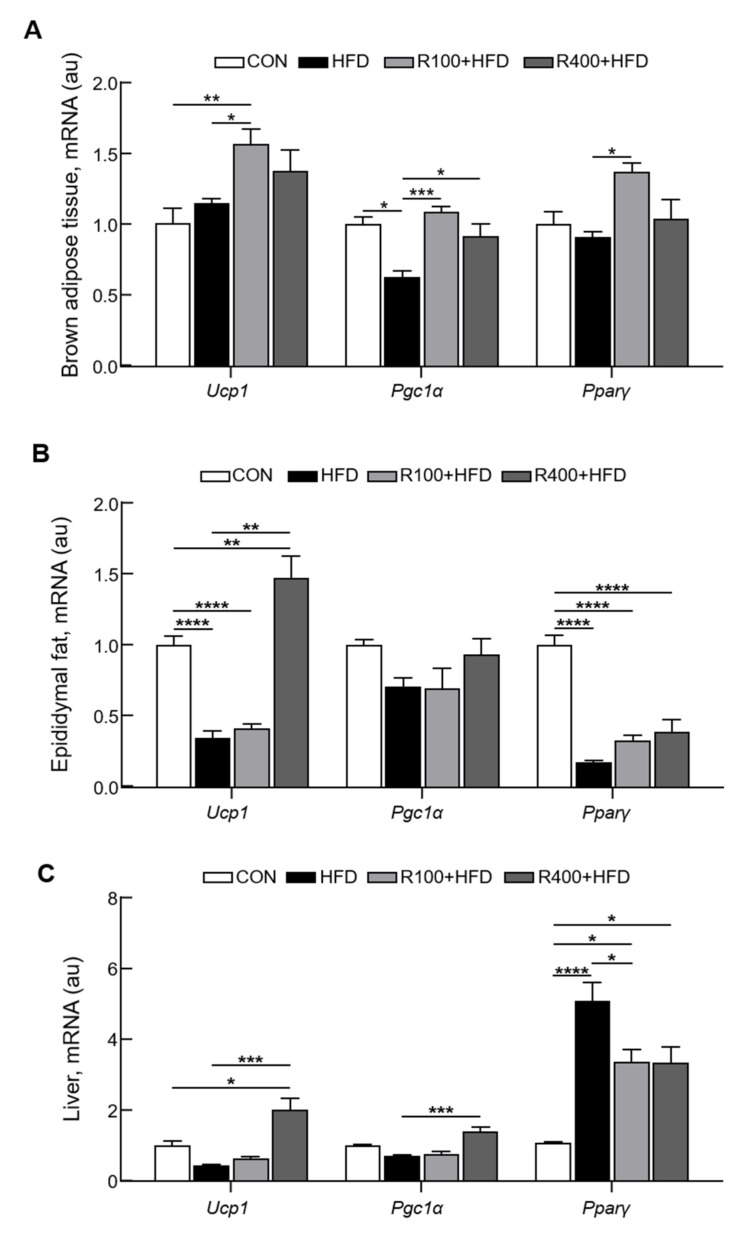
Oral feeding of *R. fasciculatum* significantly alters thermogenesis genes expression. Standard C57BL/6 mice (5 weeks old) were fed with chow diet (CON), high-fat diet (HFD, 45 kcal% fat diets), 100 mg/kg of *R. fasciculatum* (RF) extract mixed with HFD (R100 + HFD), or 400 mg/kg of *R. fasciculatum* (RF) extract mixed with HFD (R400 + HFD) for 12 weeks. Before sacrifice, mice were subjected to fasting for 8 h. (**A**) The mRNA expression levels of uncoupling protein 1 (UCP1), peroxisome proliferator-activated receptor gamma coactivator 1-alpha (PGC1α), and peroxisome proliferator-activated receptor γ (PPARγ) from brown adipose tissue (BAT) (*n* = 5–7). (**B**) The mRNA expression levels of UCP1, PGC1α, and PPARγ from epididymal fat (*n* = 4–7). (**C**) UCP1, PGC1α, and PPARγ mRNA expression level in the liver (*n* = 3–7). Data are expressed as mean ± standard error of the mean (S.E.M.). Statistical differences were determined using one-way ANOVA, followed by Tukey’s post-hoc test: * *p* < 0.05, ** *p* < 0.01, *** *p* < 0.001, **** *p* < 0.0001.

Oral supplementation could affect the signals associated with lipid catabolism, lipolysis, mitochondria biogenesis, and uncoupling protein 1 (UCP1)-positive cells in the adipose tissues and contribute to managing obesity and metabolic syndrome [18]. The qPCR was performed to examine if RF feeding could regulate the lipid catabolism genes, including thermogenic signaling in the BAT, WAT, and liver. We observed a significant increase in gene mRNA levels related to BAT activation and functions in the RF-fed group, such as UCP1, PGC1α, and PPARγ, but not in the chow and HFD group (Figure 4A). Moreover, RF feeding with HFD showed elevated mRNA levels of UCP1 and PPARγ in WAT (epididymal fat) compared with the control or HFD groups (Figure 4B). In the liver, the mRNA levels of UCP1 and PGC1α were significantly higher in the R400 + HFD group compared with other groups. Interestingly, the mRNA levels of PPARγ in the HFD group were higher than in the R100 + HFD and R400 + HFD groups (Figure 4C). These results suggest that RF feeding stimulates a thermogenic signaling cascade in adipose tissue.

### 2.5. Afzelin Directly Elevates UCP-1 Transcription in Primary Cultured Adipocytes Derived from BAT

**Figure 5:**
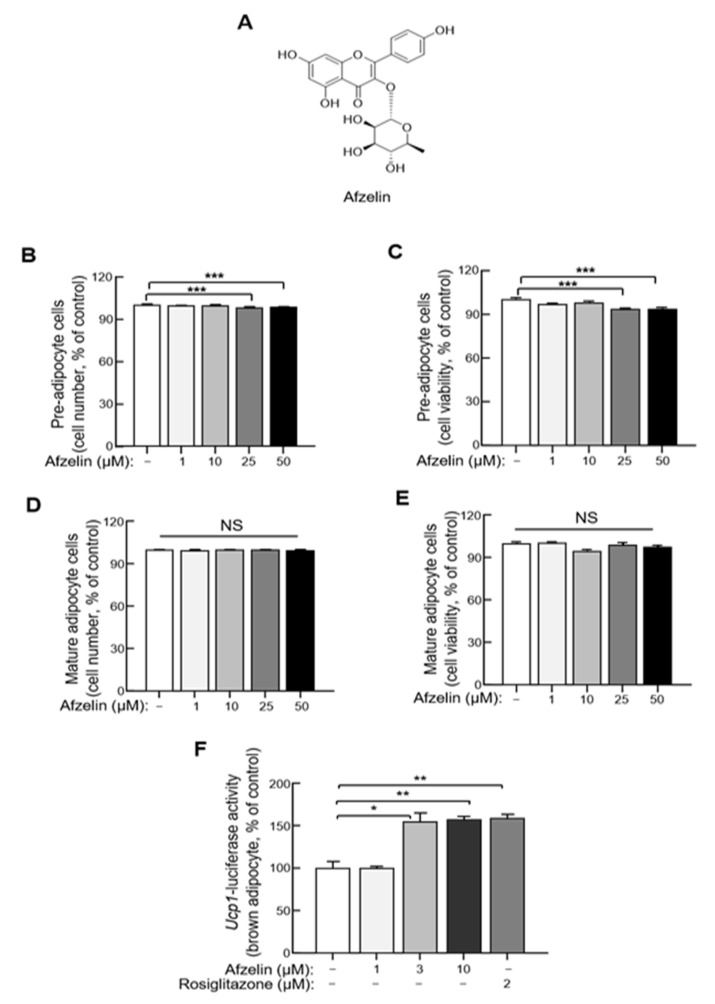
Effects of afzelin on UCP1 expression in differentiated UCP1-luciferase brown adipocytes. (**A**) The chemical structure of afzelin. The isolation of stromal vascular fraction (SVF) and immortalization was performed from brown adipose tissue of UCP1-luciferase reporter mice. (**B**) Immortalized UCP1-luciferase brown preadipocytes survival rate was measured using CCK assay. (**C**) The preadipocyte cell viability was determined by MTT assay (*n* = 6). (**D**) CCK assay proceeded mature adipocyte cells. (**E**) Mature adipocyte viability was compared with control by MTT assay (*n* = 6). (**F**) Luciferase activity was measured in differentiated UCP1-luciferase brown adipocytes treated with 1–10 μM of afzelin for 1 day (*n* = 3). Data are expressed as mean ± standard error of the mean (S.E.M.). Statistical differences were determined using one-way ANOVA, followed by Tukey’s post-hoc test: * *p* < 0.05, ** *p* < 0.01, *** *p* < 0.001.

Afzelin is a significant compound of RF with several therapeutic effects, including anticancer, renal-protective, and neuroprotective effects [17,19,20] (Figure 5A). To evaluate the cell-autonomous effects of afzelin on adipocyte thermogenesis, we isolated and immortalized brown adipocytes from Ucp1 luciferase transgenic mice showing luminescence activity when UCP1 transcription was induced [21,22]. When the cytotoxicity of afzelin was tested with the cell counting kit (CCK) and MTT assays, concentrations of >25 µM showed toxicity in the brown preadipocytes but not in mature adipocytes (Figure 5B–E). Hence, the concentration without cytotoxicity was determined, and the effect on UCP1-luciferase within the non-cytotoxic level was tested. UCP1-luciferase activity significantly increased at 3 µM of afzelin, comparable with rosiglitazone, a well-known UCP1 activator (Figure 5F). These results indicate that afzelin has a cell-autonomous effect on UCP1 transcription in brown adipocytes.

## 3. Discussion

Our study showed that the chronic feeding of RF significantly reduced HFD-induced obesity and improved metabolic-syndrome-related characteristics, including blood lipid profile, glucose tolerance, and hepatic steatosis. Despite this anti-obesity effect, RF was also associated with an increased food intake and reduced feeding efficiency, demonstrating that its effect results from regulating energy expenditure. Among the various factors controlling energy expenditure, thermogenesis contributes to the RF-mediated anti-obesity effect evidenced by significantly increasing body temperature in RF-fed mice during the 48 h cold exposure study. RF feeding also upregulated thermogenesis-related genes, including UCP1 or PGC1α, in BAT and WAT. Additionally, the treatment of afzelin notably increased UCP1-luciferase activity in primary cultured and immortalized brown adipocytes, suggesting its efficacy in elevating UCP1 transcription. Hence, we assume that chronic RF feeding partially improves obesity and related metabolic syndrome due to elevated thermogenic signaling in adipose tissues. A double-blind clinical trial also revealed that the consumption of tablets consisting of RF and *Cornus officinalis* (CO) mixture for 12 weeks significantly reduced body fat percentage and its mass in Korean overweight women [16]. In this study, we used 100 mg/kg or 400 mg/kg of RF with HFD for oral feeding to mice. According to Nair and Jacob [23], 100 mg/kg of RF administration to mice can be converted to 7.12 mg/kg of human, and an adult man can consume 427.2 mg (60 kg person) a day to reduce body weight. This consumption of RF is practical for daily intake, and RF can be suggested to be utilized as a source of functional foods for anti-obesity.

Although it appears that the elevated thermogenic signaling contributes to the RF-mediated anti-obesity effect, more studies are necessary to elucidate molecular mechanisms underlying the regulation of energy balance by RF. An in vivo study revealed that RF and CO mixture reduced HFD-induced weight gain, causing reduced abdominal visceral fat and hepatic steatosis [15]. In addition, the combination treatment of RF and CO suppressed 3T3-L1 adipocyte differentiation [15]. As a mechanism, the mixture downregulated the mRNA levels of adipogenesis-related genes in abdominal fat and 3T3-L1 adipocytes, such as CCAAT/enhancer-binding protein alpha, fatty acid-binding protein 4, PPARγ, and sterol regulatory element-binding protein [15]. Nevertheless, it is difficult to compare the combination treatment with the RF-only treatment directly; RF supplementation did not affect mRNA levels of PPARγ, a master transcription factor for adipogenesis and lipogenesis, in BAT and WAT. Thus, we surmise that the RF-mediated anti-obesity effect may not be derived from the decrease in adipogenesis signaling.

Afzelin is also a phenolic compound discovered in *Nymphaea odorata* (American white waterlily) [24,25]. Although its effects on obesity have not been elucidated, some studies indicated that afzelin suppressed UV-mediated generation of intracellular reactive oxygen species and cytotoxicity in HaCaT human keratinocytes. Additionally, it reduced UVB-induced apoptosis and exhibited anti-inflammatory properties by suppressing interleukin-6, tumor necrosis factor-α, and prostaglandin E2 in human keratinocytes [24,26]. Another study using 3T3-L1 adipocytes demonstrated that afzelin treatment moderately decreased adipocyte differentiation partly due to the downregulation of genes associated with adipogenesis, including C/EBPα and FAS [27]. The in vitro experiment we performed using brown adipocytes demonstrated that afzelin treatment (3 and 10 µM) elevated UCP1-luciferase activity without altering preadipocyte and mature adipocyte cell numbers. This result suggests that afzelin induces thermogenic signaling without affecting cell proliferation. Although these experiments were performed using brown adipocytes, it is necessary to further study the effects of afzelin on signaling pathways of white adipocyte metabolism, inflammation, and oxidative stress.

In summary, our study revealed that RF extracts stimulate thermogenic programming in BAT and WAT, resulting in its anti-obesity effect. The underlying mechanisms include but are not limited to the afzelin-mediated alteration in the mRNA expression of genes associated with thermogenic signaling. Hence, RF extracts may be incorporated into a pharmaceutical compound that prevents obesity and obesity-related metabolic disorders.

## 4. Materials and Methods

### 4.1. Animals and Experimental Design

C57BL/6 mice (male, 4 weeks old, *n* = 32) were obtained from Saeron Bio Inc. (Uiwang, Korea). The animal room, an infection-free housing condition, was maintained at constant temperature (24 °C ± 2 °C), humidity (50% ± 5%), and light interval (12 h); the animals were kept two to four mice per cage and fed diets and drinking water freely. After the initial 1 week of feeding the chow diet in the form of a pellet for adaptation, all mice were classified as control diet group (CON, chow, *n* = 8), HFD-induced obese group (HFD, 45 kcal% fat, D12451, Research Diets, New Brunswick, NJ, USA, *n* = 8), low dose of RF extract mixed with HFD-fed group (R100 + HFD, 100 mg/kg of RF, 45 kcal% fat, *n* = 8), and high dose of RF extract mixed with HFD-fed group (R400 + HFD, 400 mg/kg of RF, 45 kcal% fat, *n* = 8) by weight randomization. Next, RF was prepared via freeze-drying and then mixed with HFDs. Mice were fed these diets *ad libitum* for 16 weeks. Lastly, the body weight was measured weekly, and food intake was performed for a week each month. The animal ethics committee of the Korea Institute of Science and Technology approved all procedures adopted (Approval No. KIST-2019-048).

### 4.2. Biochemical Assays

According to the manufacturer’s instructions, the TC in plasma was measured using pharmaceutical enzymatic kits (Asan Pharm, Seoul, Korea). Free fatty acids in plasma were assessed with acyl-CoA synthetase–acyl-CoA oxidase with the NEFA-HR (non-esterified fatty acids) reagent according to the manufacturer’s instruction (Wako, Tokyo, Japan). We intraperitoneally injected 10 mL glucose per kg of body weight into experimental mice after 8 h fasting for the glucose tolerance test. Finally, the blood glucose was measured from mice’s tails at 0, 15, 30, 60, and 120 min after the glucose injection.

### 4.3. Oil Red O Staining

Immediately after excision, the liver tissue was immersed in 30% sucrose (Samchun, Seoul, Korea). Next, the liver was cut into frozen sections and air-dried on the slides. Next, formalin was used for fixation, and the slides were rinsed with water and 60% isopropanol. Afterward, the tissue was stained with freshly prepared oil red O working solution and rinsed with 60% isopropanol. Finally, the nuclei were stained with Mayer’s hematoxylin, the tissue was rinsed with distilled water, and the coverslip was placed on aqueous mounting media.

### 4.4. Body Temperature Measurements

Using rectal thermometry is a standard method for measuring body temperature in mice [28,29]. First, we kept the mice in a 4 °C cold room for 2 days. Then, the decrease in mice’s body temperatures in a cold room was measured every 0, 0.5, 1, 2, 3, 17, 19, 21, 23, 25, 27, 42, 44, 46, and 48 h by inserting a small-diameter temperature probe into the anus (Thermometer DT-610B, CEM). A depth of >2 cm was inserted into mice in a cold room with a 12 h light/dark cycle and free access to food and water.

### 4.5. Total RNA Isolation and cDNA Synthesis

Additionally, total RNA was homogenized and extracted from the BAT, WAT, and liver using TRIZOL reagent according to the manufacturer’s instructions. RNase-free DNase removed DNA contamination, and then it was reprecipitated in ethanol to prevent phenol contamination. After the chloroform was added to the extract, it was left at room temperature, and then centrifuged at 12,000 rpm and 4 °C for 15 min. The supernatant was taken, and an aliquot amount of isopropanol was added. The mixture was left to stand at ambient temperature and centrifuged at 12,000 rpm and 4 °C for 10 min. Then, 70% ethanol was added to the pellet, and it was centrifuged at 12,000 rpm for 5 min at 4 °C for washing. After washing twice, the pellet was dissolved in diethylpyrocarbonate-treated water, and the concentration was measured. An aliquot amount of RNA was pooled in each group to standardize individual differences. For cDNA synthesis, SuperScript^®^III First-Strand Synthesis System for RT-PCR Kit (CAT. 18080-051, Invitrogen by Thermo Fisher Scientific Inc., Waltham, MA, USA) was used to reverse-transcribe total RNA. It was then diluted 5-fold in nuclease-free water, stored at −70 °C, and used as a template for PCR.

### 4.6. Isolation and Structural Identification of Major Compound from RF

The dried leaves of RF (1.2 kg) were extracted twice with 30% EtOH two times at room temperature and then filtered. The filtrate was evaporated under vacuum at 40 °C to obtain an EtOH extract (303 g), which was suspended in distilled water (4 L), followed by partitioning with *n*-hexane (1.5 g), CH_2_Cl_2_ (4.0 g), EtOAc (28.0 g), and *n*-BuOH (23.5 g). The EtOAc soluble fraction (28.0 g) was separated by silica gel column chromatography using gradient mixtures as eluents (chloroform:MeOH; 100:0, 95:5, 90:10, 80:20, 70:30, 50:50, 30:70, 0:100; F001–011). F009 was purified by preparative HPLC (column: YMC-Pack ODS-A, 5 μm, 250 × 20 mm I.D., Japan, 8 mL/min, MeCN-H_2_O, 5:95 to 80:20, 60 min) to yield compound (Supplementary Figure S1A,B). The chemical structure was identified as afzelin, based on the results of spectroscopic data including mass [1], H-NMR [13], and C-NMR [30]. The HPLC results provided specific information on the major compound, afzelin, in RF (Supplementary Figure S2).

### 4.7. Isolation and Immortalization of Ucp1 Luciferase Adipocytes

Professor Shingo Kajimura at the University of California San Francisco provided the *Ucp1* luciferase reporter mice (Thermo mouse) [22]. The Thermo mouse was backcrossed to C57BL/6J mice for nine generations. The isolation of stromal vascular fraction (SVF) and immortalization was performed as described [22]. SVF was isolated from interscapular BAT of Thermo mouse (5-week-old male). For immortalization, the cells infected with a retrovirus expressing large T antigen (pBabe SV40 Large T antigen; Addgene) were selected by puromycin (2 μg/mL).

### 4.8. Cell Culture and Differentiation

Dulbecco’s modified Eagle’s medium (DMEM) and penicillin/streptomycin (P/S) was purchased from Hyclone (Logan, UT, USA), and the fetal bovine serum (FBS) was obtained from Gibco (Thermo Fisher Scientific, Waltham, MA, USA). The translucent PET used for cell culture was acquired from Sarstedt (Nümbrecht, Germany). CCKs were obtained from Dojindo Molecular Technologies (Kumamoto, Japan). Immortalized *Ucp1* luciferase brown preadipocytes were cultured in DMEM, 10% FBS, and 1% P/S. Upon 100% confluence (day 0), the cells were differentiated in the presence of 10% FBS, 5 μg/mL insulin, 1 nM T3, 2 μg/mL dexamethasone, 125 μM 3-isobutyl-1-methylxanthine, and 125 μM indomethacin for 2 days. Then, the medium containing only insulin and T3 was changed every 2 days.

### 4.9. Cell Viability Assay

Immortalized Ucp1 luciferase brown preadipocytes were plated in 96-well culture plates (5 × 104 cells/mL) to determine cell viability. The cells were differentiated into mature adipocytes for 6 days or not. Next, the preadipocytes and mature adipocytes were treated with various concentrations of afzelin for 24 h. After the incubation, CCK (1/10 volume of the culture medium) or MTT (final 0.5 mg/mL) solution was added and incubated for an additional 2 h (*n* = 6). The CCK assay was calculated as optical density using an ELISA plate reader (450 nm; SpectraMax i3; Molecular Devices, San Jose, CA, USA). To examine the MTT assay, purple formazan was dissolved in 100 μL of dimethyl sulfoxide. After incubation for 10 min, the absorbance at 570 nm was measured in the plate reader.

### 4.10. Luciferase Assay

Additionally, immortalized *Ucp1* luciferase brown preadipocytes were seeded in 12-well plates (8 × 10^4^ cells/mL). Then, at 100% confluence, the cells were differentiated into mature adipocytes for 6 days (*n* = 3). Next, the cells were lysed in Bio-Glo™ Luciferase Assay System (Promega, Madison, WI, USA). Finally, the luciferase activity was determined using a GloMax^®^ Explorer Multimode Microplate Reader (Promega).

### 4.11. Statistical Analysis

All experimental values are shown as mean ± standard error of the mean and evaluated with one-way ANOVA using Tukey’s test. The statistical analysis was performed using the GraphPad PRISM software (GraphPad PRISM Software Inc., version 8, San Diego, CA, USA); *p* values of <0.05 were considered significant.

## Data Availability

Not applicable.

## Supplementary Materials

The following supporting information can be downloaded online, Figure S1: Structure elucidation and identification of afzelin from *Ribes fasciculatum* extract Isolation scheme of compound from *R. fasciculatum* (A), HPLC profile of *R. fasciculatum* Extract and afzelin (B), Mass spectrum of afzelin from the *R. fasciculatum* (C), Figure S2: NMR spectral data of afzelin from *Ribes fasciculatum* extract, ^13^C-NMR (CD_3_OD, 175 MHz, DMSO-d_6_), (D), ^1^H-NMR (CD_3_OD, 700 MHz, DMSO-d_6_), (E).
