# Supplementary material for: Ribes fasciculatum Ameliorates High-Fat-Diet-Induced Obesity by Elevating Peripheral Thermogenic Signaling
Source: Molecules. 2022 Mar 2;27(5):1649. doi: 10.3390/molecules27051649 (PMC8911937; doi:10.3390/molecules27051649)
Supplement: Supplementary file 1 [file molecules-27-01649-s001.zip › molecules-1570985-supplementary.pdf]

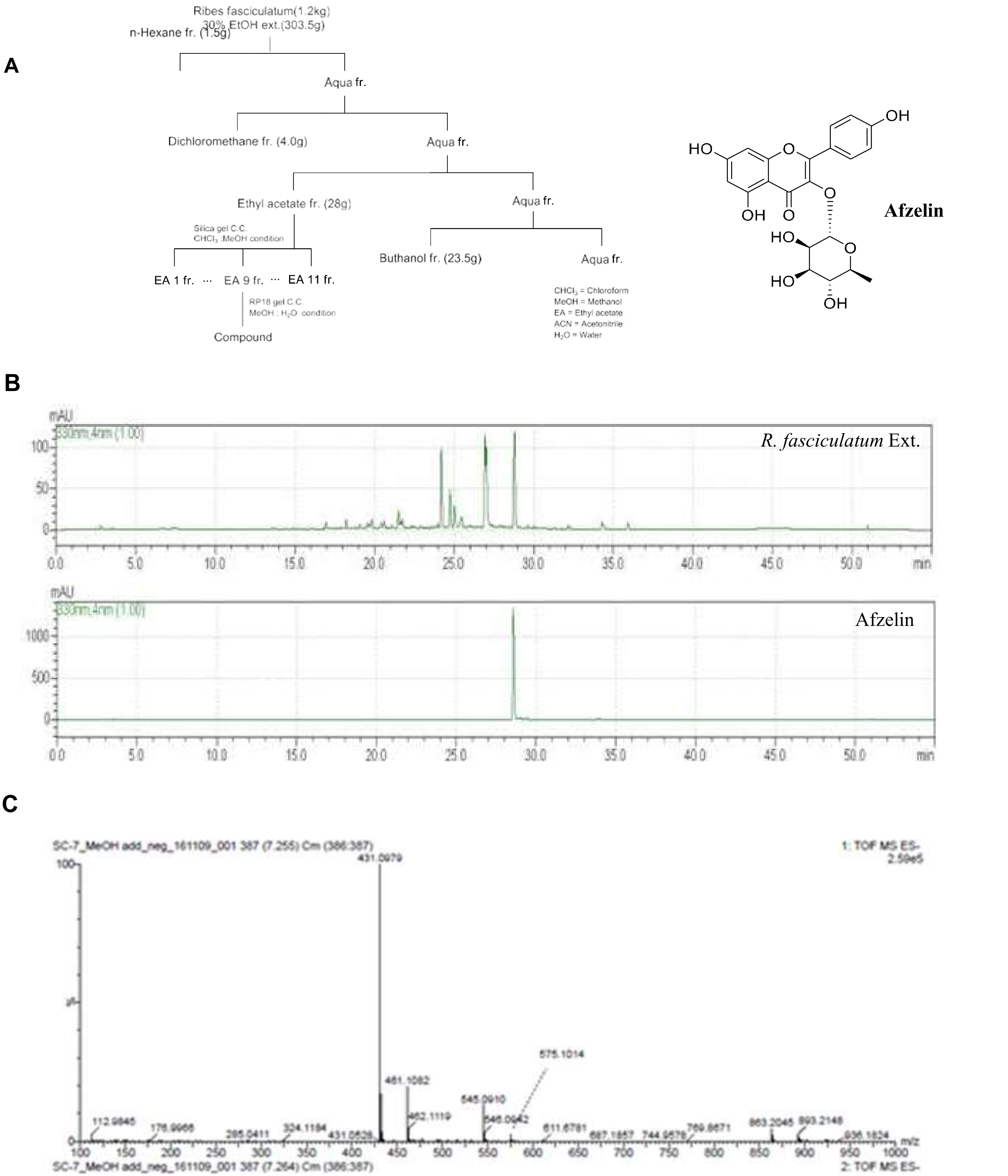

Figure S1. Structure elucidation and identification of afzelin from *Ribes fasciculatum* extract Isolation scheme of compound from *R. fasciculatum* (A), HPLC profile of *R. fasciculatum* Extract and afzelin (B), Mass spectrum of afzelin from the *R. fasciculatum* (C).

D

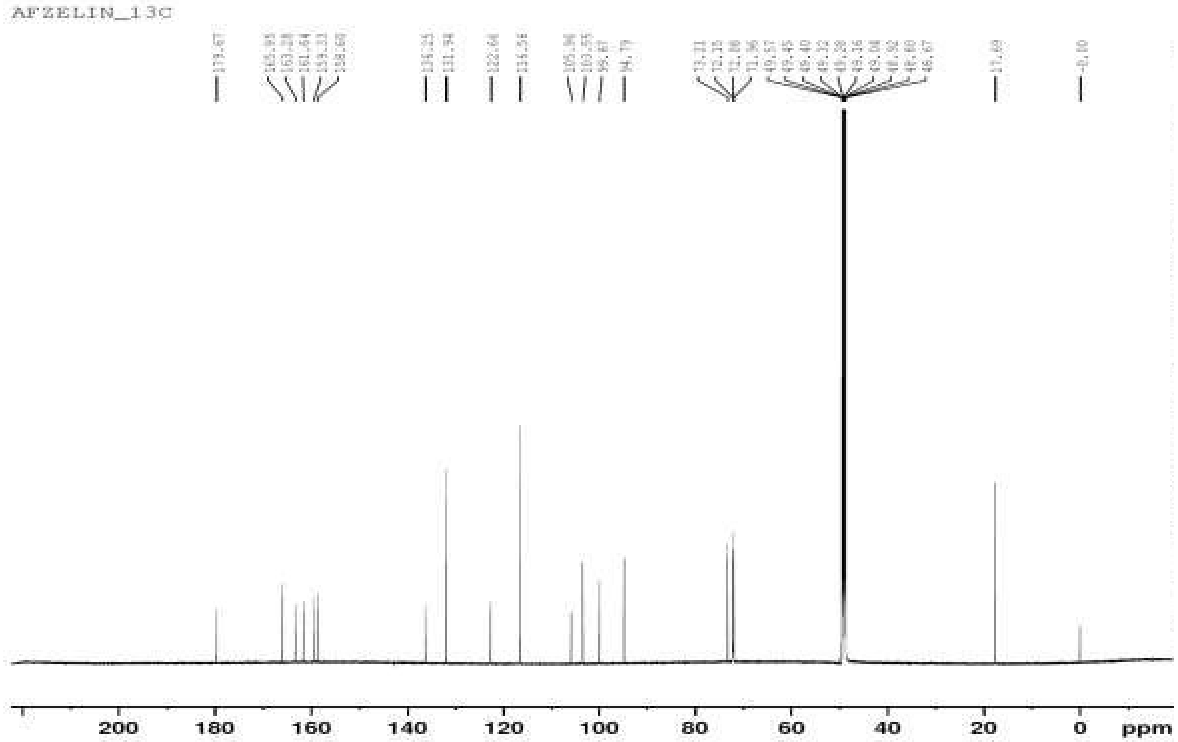

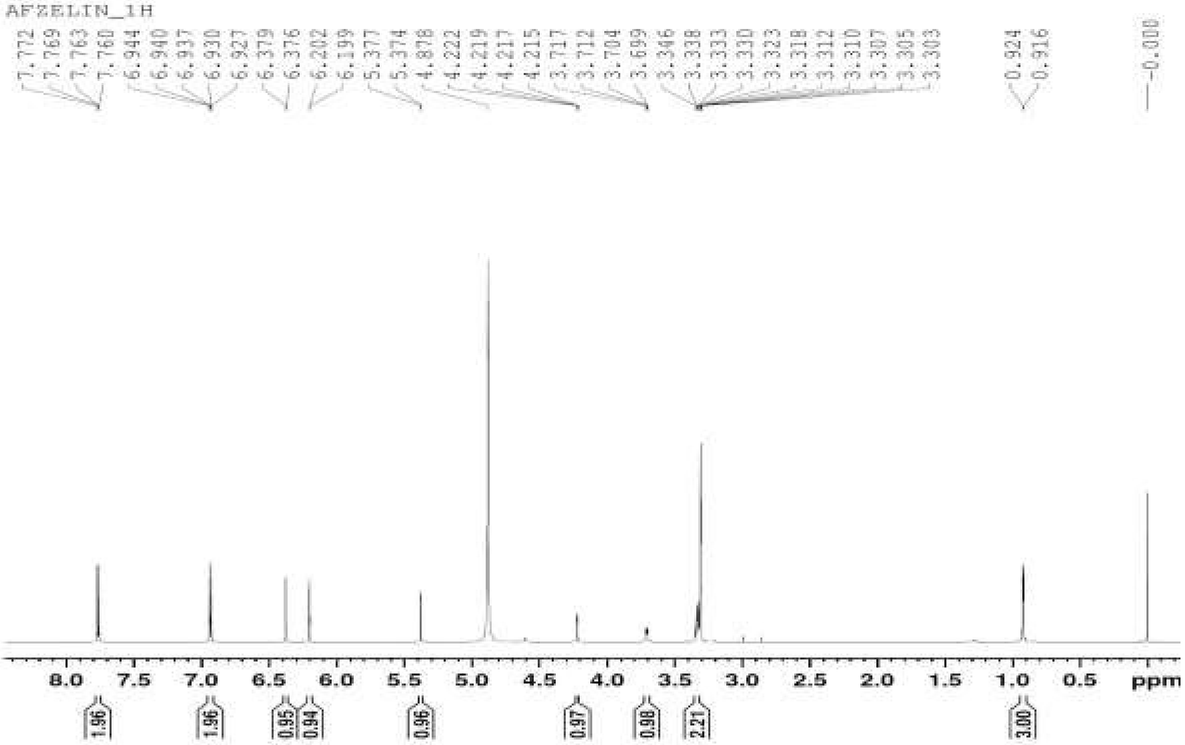

E

Figure S2. NMR spectral data of afzelin from *Ribes fasciculatum* extract, ^{13}C-NMR (CD_3OD, 175 MHz, DMSO-d_6), (D), ^{1}H-NMR (CD_3OD, 700 MHz, DMSO-d_6), (E).
